# Assessment of Selected Clinical Chemistry Analytes Among Hypertensive Patients at Wallaga University Referral Hospital, Nekemte, Oromia, Western Ethiopia: A Comparative Cross‐Sectional Study

**DOI:** 10.1002/hsr2.72921

**Published:** 2026-07-29

**Authors:** Amanuel Hika, Sintayehu Asaye Biya, Temam Ibrahim Husen, Negesse Bokona Rufe, Hundesa Emana Urgesa, Bedasa Addisu

**Affiliations:** ^1^ Department of Clinical Chemistry, School of Medical Laboratory Sciences, Institute of Health Sciences Wallaga University Nekemte Ethiopia; ^2^ Department of Clinical Chemistry, School of Medical Laboratory Sciences, Institute of Health Jimma University Jimma Ethiopia; ^3^ Department of Medical Laboratory Science, College of Health Science Salale University Fitche Ethiopia; ^4^ Department of Medical Biochemistry, School of Medicine, College of Health and Medical Sciences Haramaya University Harar Ethiopia; ^5^ Department of Medical Laboratory Science, College of Medicine and Health Sciences Debre Berhan University Debre Berhan Ethiopia

**Keywords:** clinical chemistry analytes, hypertension, Nekemte

## Abstract

**Background and Aims:**

Hypertension (HTN) is a long‐term condition marked by consistently high arterial blood pressure. It is a leading contributor to cardiovascular diseases and early mortality around the globe. It increases the risk of ischemic heart disease, stroke, chronic kidney disease (CKD), and dementia. So far, several studies have been conducted, but to the authors’ knowledge, there is a scarcity of similar studies done on the selected analytes in our setting, which can be an important input for the early detection of disturbance in specific parameters. So, this study aims to assess selected Clinical Chemistry analytes in hypertensive patients.

**Methods:**

A comparative cross‐sectional study was conducted at Wallaga University Referral Hospital among 105 hypertensive patients and 105 non‐hypertensive controls from June 2 to August 20, 2022. Participants were interviewed using structured questionnaires about their socio‐demographics and measured for their anthropometric characteristics. Then, five milliliters (5 mL) of blood samples were collected and analyzed for fasting blood sugar (FBS), serum electrolytes, creatinine, uric acid, and lipid profiles using a Cobas c‐311 clinical chemistry analyzer. Data were entered into Epi Data version 4.6 and analyzed using SPSS version 26. A *p*‐value of < 0.05 was considered statistically significant at a 95% confidence level.

**Results and Conclusion:**

The average age of the hypertensive and control groups was 54.69 ± 12 years and 44.82 ± 12.9 years, respectively. Hypertensive patients had significantly lower mean ± SD levels of serum creatinine, total cholesterol (TC), LDL cholesterol (LDL‐C), HDL cholesterol (HDL‐C), and sodium (*p* < 0.05) compared to non‐hypertensive participants. In contrast, they had significantly higher mean ± SD levels of fasting blood sugar (FBS) and triglycerides (TG). In this study, we found that the hypertensive group faced an increased risk of clinical chemistry analyte changes over time. Therefore, regular monitoring of Clinical Chemistry parameters is crucial for patients with hypertension.

**Abbreviations**
AOR: Adjusted Odds ratio
BMI: Body mass index
CRO: Crude Odds ratio
FBG: Fasting blood glucose
HDL‐C: High‐density lipoprotein cholesterol
HTN: Hypertension
LDL‐C: Low‐density lipoprotein cholesterol
Mean ± SD: Mean ± standard deviation
mg/dL: Milligram per deciliter
SPSS: Statistical Package for Social Science
TC: Total cholesterol
WHO: World Health Organization
WURH: Wallaga University Referral Hospital
Mean ± SD: Mean ± standard deviation

## 1 Introduction

Hypertension (HTN) is a long‐term condition marked by high arterial blood pressure (BP). It hinders the heart's ability to pump blood effectively through the vessels [1]. Blood pressure spikes cause damage to the vessel wall, which results in fat buildup and wall thickening. If hypertension is neglected, it can lead to more severe diseases or complications, such as angina, myocardial infarction, heart failure, and kidney failure, by decreasing the blood flow to the heart, brain, kidneys, and extremities [2].

The World Health Organization (WHO) estimates that approximately 62% of cardiovascular disease (CVD) and 49% of ischemic heart disease worldwide are due to hypertension. According to the Global Report on HTN Epidemiology, hypertension accounts for 13% of the world's deaths, with 48% of deaths due to cardiovascular disease [3]. The prevalence of hypertension is estimated to be about 30.8% in Africa and 30.0% to 31.1% in sub‐Saharan Africa. Hypertension is now a serious problem in groups previously thought to be low risk, such as rural populations, poor households, and young people [4].

A recent study conducted on adult hospital outpatients in Addis Ababa [5] and Nekemte town found the prevalence of hypertension at 34.7% and 34.9%, respectively [6]. Various studies conducted showed alterations in biochemical parameters among hypertensive and normotensive patients. A case‐control study done in India [7] and Iran [8] found significantly higher values of lipid profile among hypertensive patients. Another study done in Nigeria reported that hypertensive patients’ and healthy individuals’ blood electrolytes are more significantly different [9].

The primary risk factors for hypertension include a personal history of cardiovascular disease (CVD), stroke, transient ischemic attacks, diabetes, dyslipidemia, chronic kidney disease, smoking, unhealthy diet, alcohol consumption, physical inactivity, psychosocial factors, a history of depression, and a family history of hypertension [10, 11, 12].

Diabetes damages the small blood vessels in your body, causing the walls of the blood vessels to stiffen and resulting in atherosclerosis, which can lead to trouble, including blood vessel damage, heart attack, and kidney failure. Patients with diabetes mellitus also have higher peripheral arterial resistance due to vascular remodeling and higher body fluid volume, which are linked to hyperglycemia and insulin resistance‐induced hyperinsulinemia [13, 14].

The kidney, the main target of organ damage in hypertension and long‐term elevations of blood pressure (BP), even within the normal range, can induce early renal damage. Inadequate management of hypertension is linked to elevated serum creatinine levels, which are a sign of chronic renal impairment. Furthermore, as a low renal blood flow will promote urate reabsorption, a decrease in renal blood flow that occurs with hypertension may also be the cause of an increase in serum uric acid [15, 16].

Too much salt intake makes the body hold extra water to “wash” the salt from the body; the added water puts stress on the heart and blood vessels, causing blood pressure to rise. Potassium plays a key role in regulating sodium levels in your body. When potassium levels are low, the kidneys retain more sodium in the body, which can lead to increased blood pressure [17].

Dyslipidemia is another risk factor associated with increased blood pressure and increased levels of low‐density lipoprotein, total cholesterol, and triglycerides in the blood, accounting for the highest morbidity and mortality. In contrast, low levels of high‐density lipoproteins are a risk factor for death from cardiovascular disease [18].

Hypertension can be managed through medication; however, data show that, on average, fewer than 50% of adults with hypertension receive blood pressure‐lowering drugs. Lifestyle modifications are also crucial as the first line of treatment [19, 20].

Generally, HTN is a globally recognized threat to social and economic development, with premature morbidity and mortality. Although many different studies have been performed on hypertensive patients, the burden of hypertension can vary from person to person and by geographical location, depending on age, environment, and lifestyle‐related factors. Accordingly, the alteration in biochemical analytes among hypertensive patients varies across populations and geographical locations due to differences in lifestyle and other risk factors. Limited research has been conducted on alterations in clinical chemistry analytes among hypertensive and non‐hypertensive individuals in Ethiopia. This study aims to assess these analytes among hypertensive patients at Wallaga University Referral Hospital (WURH) in Nekemte, Western Ethiopia.

## 2 Participants and Materials

### 2.1 Study Setting and Period

The study was conducted in Wallaga University Referral Hospital (WURH), located 331 kilometers from Addis Ababa, the capital of Ethiopia, from June 2 to August 20, 2022. WURH was established in 2017 as the only teaching and referral hospital in western Ethiopia. The hospital has surgical, gynecology & obstetric, internal medicine, and pediatric departments. Each department has its inpatient, outpatient, and referral clinics. It provides different health services for more than 1,756,952 people [21].

### 2.2 Study Design

A comparative cross‐sectional study was conducted to assess selected clinical chemistry analytes among hypertensive patients on follow‐up during the study period.

### 2.3 Populations

#### 2.3.1 Source Populations

All hypertensive patients on follow‐up in Wallaga University Referral Hospital from May 09 to July 29, 2022.

#### 2.3.2 Study Population

All confirmed Hypertensive outpatients on follow‐up who are attending WURH and all healthy control groups who are visiting the outpatient department (OPD) during the study period.

#### 2.3.3 Inclusion Criteria

Patients with hypertension, study participants who voluntarily participated, and healthy non‐hypertensive subjects as the control group were included in this study.

#### 2.3.4 Exclusion Criteria

- **From Cases**

∘ Severely ill patients
∘ Participants who have a history of renal disease, pregnant mothers with new‐onset hypertension, heart failure, and diabetes were excluded.

- **From control**

∘ Severely ill patients
∘ Participants who have a history of renal disease, pregnant mothers with new‐onset hypertension, heart failure, and diabetes were excluded.

### 2.4 Study Variables

#### 2.4.1 Dependent Variable

Selected clinical chemistry analytes (Fasting blood glucose, serum electrolytes, creatinine, Uric acid, and lipid profile).

#### 2.4.2 Independent Variables

Socio‐demographic characteristics, body mass index (BMI), duration of HTN, lifestyle‐related factors (smoking status, alcohol intake), and medications were the explanatory variables of this study.

### 2.5 Sample Size Determination and Sampling Technique

#### 2.5.1 Sample Size Calculation

The sample size was determined using the two‐population sample size method with G Power version 3.1.9.7, based on the mean and standard deviation from a study comparing hypertensive and non‐hypertensive groups. The software calculated the sample size, resulting in a total of 210 participants: 105 from the hypertensive group and 105 from the normotensive unexposed group.

#### 2.5.2 Sampling Technique

A consecutive sampling method was employed to select 105 hypertensive outpatients who visited the hypertensive outpatient department during the study period, along with 105 healthy participants visiting the outpatient department.

### 2.6 Measurement and Data Collection

#### 2.6.1 Data Collection Techniques

The hypertensive patients and healthy individuals were interviewed with a questionnaire that was originally prepared in English and then translated into the Afan Oromo language to collect sociodemographic and other related clinical data.

The trained nurses conducted anthropometric measurements with participants wearing light clothing and no shoes, using a scale placed on a level floor. Body weight was recorded to the nearest kilogram with a mechanical scale (Camry), and height was measured to the nearest 0.1 cm in a standing position using a portable stadiometer (SECA 213). BMI was calculated by dividing weight (kg) by height squared (m^2^). Weight classifications were based on WHO guidelines: underweight (< 18.5 kg/m^2^), normal (18.5–24.99 kg/m^2^), overweight (25.0–29.99 kg/m^2^), and obese (≥ 30 kg/m^2^). A BMI of 25.0 kg/m^2^ or higher indicates overweight or obesity.

Waist circumference was measured once to the nearest centimeter using a constant tension tape (SECA 201), positioned directly on the skin or over light clothing at the midpoint between the lower edge of the last rib and the iliac crest along the midaxillary line. According to WHO criteria, abdominal obesity is defined as a waist circumference of 94 cm for men and 80 cm for women, or a waist‐to‐hip ratio (WHR) of 0.90 in men and 0.85 in women. Participants with a normal BMI (18.5–24.99 kg/m^2^) but with abdominal obesity were classified as having normal‐weight central obesity [22].

The impact of physical inactivity on global health issues was examined, with physical activity levels assessed and categorized using the International Physical Activity Questionnaire (IPAQ) based on 1 week or 7 days of activity among the study groups [23]. Additionally, medical records of hypertensive participants were reviewed to gather clinical information and the duration of hypertension.

#### 2.6.2 Laboratory Analysis

##### 2.6.2.1 Specimen Collection and Processing

A 5 mL fasting blood sample was collected from the study participants, and the sample was left to clot at room temperature for 30 min, then centrifuged for 4 min at 4000 revolutions per minute (rpm) to separate the serum from the cellular components. Then the serum sample was analyzed for fasting blood glucose, serum electrolytes, uric acid, creatinine, and lipid profiles using the Cobas c 311 analyzer. The machine utilizes the spectrophotometric principle for fasting blood glucose, serum electrolytes, uric acid, creatinine, and lipid profiles, while it uses the ion‐selective electrode principle for serum electrolyte analysis.

### 2.7 Data Quality Control

The Questionnaires were translated from English to Afan Oromo, and before the actual data collection, a pretest was performed on 10% of the questionnaires. Then, all required modifications were made based on the feedback from the pretest. Training and all guidance were given to data collectors to ensure the validity and completeness of the data. All laboratory tests were done by following the standard operating procedures (SOPs) and the manufacturer's instructions. The participants’ samples were analyzed only after both controls were validated, and the results were interpreted using the Westgard rule.

## 3 Data Analysis

The data were entered into Epi Data version 4.6.0 (Epi‐Data, Odense, Denmark) and analyzed using Statistical Package for Social Science (SPSS) version 26 (SPSS, Chicago, USA). In addition, the Shapiro–Wilk test and visual inspection of Q‐Q plots were used to check the normal distribution of data. Descriptive statistics were used to summarize both continuous and categorical variables. Bivariate logistic regression assessed the relationship between the dependent and independent variables. Variables that had a *p*‐value < 0.25 in the bivariable analysis were used as candidates for multivariable logistic regression. All statistical tests were two‐sided, and two‐sided p‐values less than or equal to 0.05 at a 95% confidence interval were considered statistically significant. Results were presented in tables and figures. The mean differences (SD) values of selected clinical chemistry parameters between study subjects were analyzed using an independent‐samples t‐test.

## 4 Results

### 4.1 Socio‐Demographic Characteristics of Study Participants

A total of 210 participants were enrolled in the study, achieving a 100% response rate. The average age of the participants was 54 ± 13.0 years, with 45 (42.9%) being female hypertensive patients. Most participants were married 93 (88.6%), 39 (37.1%) had no formal education, 45 (42.9%) were employed in the public or private sectors, and 69 (78.4%) had a monthly income of less than 1000 Ethiopian birr (Table 1).

**Table 1:** Socio‐demographic Characteristics of hypertensive patients and non‐hypertensive study participants at Wallaga University Referral Hospital from June 2 to August 20, 2022.

Variables		Study Participants (*n* = 210)				*p* value
		Hypertensive		Non‐hypertensive		
		*n* = 105	%	*n* = 105	%	
Age	< 50 years	38	36.2%	73	69.5%	< 0.001
	≥ 50	67	63.8%	32	30.5%	
Gender	Male	60	57.1%	57	54.3%	0.68
	Female	45	42.9%	48	45.7%	
Marital status of participants	Single	0	0.0%	6	5.7%	0.04
	Married	93	88.6%	86	81.9%	
	Divorced	0	0.0%	2	1.9%	
	Widowed	12	11.4%	11	10.5%	
Educational level of participants	No formal education	39	37.1%	20	19.0%	0.028
	Primary	17	16.2%	22	21.0%	
	secondary	22	21.0%	33	31.4%	
	higher education	27	25.7%	30	28.6%	
Occupation	gov't employee	45	42.9%	46	43.8%	0.001
	merchant	11	10.5%	18	17.1%	
	Farmer	49	46.7%	31	29.5%	
	unemployed	0	0.0%	10	9.5%	
Average monthly income level (ETB)	< 1000	19	21.6%	6	8.0%	0.02
	≥ 1000	69	78.4%	69	92.0%	

Regarding lifestyle factors, anthropometric measurements, and dietary salt habits of hypertensive patients: the majority, 101 (96.2%), did not smoke, 17 (16.2%) consumed alcohol, 6 (5.7%) were overweight, and 4 (3.8%) were obese. For non‐hypertensive participants, the majority, 102 (97.1%), did not smoke, 10 (9.5%) consumed alcohol, 5 (4.7%) were overweight, and 97 (92.4%) had normal weight (Table 2).

**Table 2:** Lifestyle factors, anthropometric, and dietary salt habits of study participants at Wallaga University Referral Hospital from June 2 to August 20, 2022.

Variables	Study participants		*p* value
	Hypertensive group	Non‐hypertensive group	
Smoking status			
Yes	4 (3.8%)	3 (2.9%)	0.70
No	101 (96.2%)	102 (97.1%)	
Smoking frequency			
Daily	2 (50%)	1 (33.3%)	0.45
Frequently	1 (25%)	2 (66.6%)	
Rarely	1 (25%)	0 (0%)	
Alcohol consumption			
Yes	17 (16.2%)	10 (9.5%)	0.15
No	88 (83.8%)	95 (90.5%)	
Alcohol drinking frequency			
Daily	4 (23.5%)	1 (10.0%)	0.39
Frequently	12 (70.6%)	8 (80.0%)	
Rarely	1 (5.9%)	1 (10.0%)	
Dietary salt consumption			
Yes	74 (70.5%)	92 (87.6%)	0.002
No	31 (29.5%)	13 (12.4%)	
Physical exercises activity			
Low	57 (63.3%)	67 (68.3%)	0.001
Moderate	33 (36.6%)	31 (31.7)	
High	15 (14.3%)	7 (6.7%)	
BMI			
Normal weight	95 (90.5%)	97 (92.4%)	0.62
Overweight	6 (5.7%)	5 (4.7%)	
Obese	4 (3.8%)	3 (2.8%)	

*P*‐value= chi‐square test

### 4.2 Age Comparison Between Hypertensive and Non‐Hypertensive Participants

From the total of 105 hypertensive patients, 67 (63.8%) were aged greater than 50 years, while only 32 (30.5%) were aged greater than 50 years from 105 non‐hypertensive patients, which indicates hypertension is significantly higher among the older group(*P* ≤ 0.001). This also implies hypertensive patients were older than the non‐hypertensive group, which was confirmed by an independent‐samples *t*‐test showing a statistically significant difference in mean age between the two groups (*t* (208) = 5.728, *p* < 0.001). Moreover, the mean age was 54.69 ± 12.02 years among hypertensive participants and 44.82 ± 12.92 years among non‐hypertensive ones (Table 3).

**Table 3:** Age Comparison between study participants at Wallaga University Referral Hospital from June 2 to August 20, 2022.

Variable	Hypertensive (*n* = 105)	Non‐hypertensive (*n* = 105)	Test statistic	*p* value
Age group, *n* (%)			*χ* ^2^	
< 50 years	38 (36.2)	73 (69.5)		< 0.001
≥ 50 years	67 (63.8)	32 (30.5)		
Age (years), Mean ± SD	54.69 ± 12.02	44.82 ± 12.92	t(208) = 5.73	< 0.001
Mean difference (95% CI)	9.87 years (6.28–12.99)			

### 4.3 Comparison of Laboratory Test Parameters Among Study Groups

Hypertensive patients showed significantly lower mean ± SD levels of serum creatinine, total cholesterol (TC), LDL cholesterol (LDL‐C), HDL cholesterol (HDL‐C), and sodium (*p* < 0.05) compared to non‐hypertensive participants. Conversely, hypertensive patients had significantly higher mean ± SD levels of fasting blood sugar (FBS) and triglycerides (*p* < 0.05). There were no significant differences in potassium and uric acid levels between the two groups (Table 4).

**Table 4:** Comparison of laboratory test parameters among study groups at Wallaga University Referral Hospital from June 2 to August 20, 2022.

Test Parameters	Study participants		*t*‐test for equality of means		
	Hypertensive	Non‐hypertensive	*p* value	95%CI of the Difference	
	Mean ± SD	Mean ± SD		Lower	Upper
Creatinine (mg/dL)	0.83 ± 0.33	0.88 ± 0.49	0.03	−0.17	0.056
Uric acid (mg/dL)	4.73 ± 1.2	4.8 ± 1.6	0.73	−0.45	0.32
FBG (mg/dL)	122.6 ± 49.5	102.6 ± 29.56	< 0.001	8.89	31.07
Total Cholesterol (mg/dL)	169.2 ± 45.3	185.2 ± 49.7	0.02	−28.9	−3.03
Triglyceride (mg/dL)	179.3 ± 58	151.7 ± 49.4	< 0.001	12.9	42.22
HDL‐C (mg/dL)	30.3 ± 24.7	51.4 ± 51.3	< 0.001	−32.08	−10.14
LDL‐C (mg/dL)	104.6 ± 42.8	120 ± 44.9	0.01	−27.34	−3.44
Sodium (mmol/L)	134.5 ± 11.9	138.7 ± 5.1	< 0.001	−6.70	−1.73
Potassium (mmol/L)	4.8 ± 0.9	5.5 ± 1.4	0.06	−0.95	1.29

### 4.4 The Laboratory Test Parameters and Associated Factors Among Study Groups

#### 4.4.1 Duration of Hypertension With Laboratory Parameters Among Hypertensive Patients

Hypertensive patients with a hypertension duration of over 5 years had significantly higher levels of fasting blood sugar (FBS), creatinine, uric acid, total cholesterol (TC), LDL cholesterol (LDL‐C), and potassium compared to those with a hypertension duration of 5 years or less. There were no significant differences in serum sodium, triglycerides (TG), and HDL cholesterol (HDL‐C) between the two groups (Table 5).

**Table 5:** Duration of hypertension with laboratory test parameters among hypertensive patients at Wallaga University Referral Hospital from June 2 to August 20, 2022.

Test parameters	Study participants		*t*‐test for equality of means		
	< 5 years (*n* = 81) Mean ± SD	≥ 5 years (*n* = 24) Mean ± SD	*p* value	95% CI	
				Lower	Upper
Creatinine (mg/dL)	0.78 ± 0.31	0.96 ± 0.37	0.02	−0.32	−0.02
Uric acid (mg/dL)	4.41 ± 0.98	5.02 ± 0.83	0.007	−1.05	−0.17
FBG (mg/dL)	123.96 ± 52.1	163.91 ± 46.01	0.001	−63.36	−16.52
Total Cholesterol (mg/dL)	169.20 ± 46.24	209.98 ± 61.13	0.001	−63.80	−17.75
Triglyceride (mg/dL)	186.38 ± 61.81	201.75 ± 59.94	0.28	−43.67	12.93
HDL‐C (mg/dL)	28.72 ± 15.53	32.74 ± 40.08	0.46	−14.79	6.74
LDL‐C (mg/dL)	108.75 ± 43.53	129.02 ± 37.72	0.04	−39.77	−0.77
Sodium (mmol/L)	132.59 ± 11.07	136.77 ± 11.44	0.11	−9.52	1.17
Potassium (mmol/L)	4.68 ± 0.91	5.15 ± 0.97	0.29	−0.90	1.02

### 4.5 Factors Associated With Abnormal Laboratory Parameters in People With Hypertension

Physical activity and alcohol consumption were found to be independent factors significantly linked to elevated creatinine and uric acid levels. Hypertensive individuals with low physical activity were 2 times more likely to have elevated creatinine and uric acid, while alcohol consumers were 5.32 and 4.34 times more likely to have elevated creatinine and uric acid, respectively (Table 6).

**Table 6:** Logistic regression analysis for factors associated with abnormal creatinine and uric acid results among hypertensive patients at Wallaga University Referral Hospital from June 2 to August 20, 2022.

Variables			Dependent variables					
			Serum creatinine			Serum uric acid		
Independent variable		COR (*P*)	AOR	*P*	COR (*P*)	AOR	*P*
	Physical activity							
	Low		3.068 (0.09)	2.15 (1.01,4.16)	0.004	3.07 (0.18)	2.28 (0.8, 5.56)	0.009
	High		1*			1*		
	Alcoholic drinking							
	Yes		7.5 (0.02)	5.32 (1.52,10.3)	0.02	3.01 (0.08)	4.34 (1.027,18.37)	0.04
	No		1*			1*		
	Smoking status							
	Yes		6.6 (0.11)	1.219 (0.01,2.9)	0.48	2.43 (0.05)	2 (0.196, 6.32)	0.56
	No		1*			1*		
	Gender							
	Male		0.76 (0.08)	0.237 (0.024,2.35)	0.22	0.78 (0.16)	0.554 (0.164, 1.88)	0.34
	Female		1*			1*		
	Age							
	≥ 50		0.55 (0.05)	2.67 (0.417,17.13)	0.29	1.83 (0.17)	3.69 (1.22, 6.18)	0.53
	< 50		1*			1*		

*Note: p* < 0.05 statistically significant, 1*=Reference category, AOR= adjusted odds ratio, COR= crude odds ratio.

Obese hypertensive individuals had a 5.9 times higher likelihood of developing hypercholesterolemia compared to those with normal weight (AOR = 5.9; 95% CI = 1.94–18.06). Furthermore, physical activity and alcohol consumption were also identified as significant independent factors influencing hypercholesterolemia and hypernatremia among hypertensive individuals. Moreover, hypertensive individuals aged > 50 were 1.95 more likely to have hypernatremia(AOR = 1.95; 0.5–4.18) (Table 7).

**Table 7:** Logistic regression analysis for factors associated with abnormal hyponatremia and hypercholesterolemia among hypertensive patients at Wallaga University Referral Hospital from June 2 to August 20, 2022.

Variables		Dependent variables					
		Total cholesterol			Sodium		
		COR (*p* value)	AOR (95% CI)	*p* value	COR (*p* value)	AOR (95% CI)	*p* value
Independent variables	Physical activity						
	Low	1.49 (0.06)	2.06 (1.03,3.09)	0.05	4.02 (0.23)	2.96 (2.13, 6.05)	0.05
	High	1*			1*		
	Alcohol drinking						
	Yes	3.42 (0.004)	2.46 (1.05,6.23)	0.05	6.3 (0.001)	4.31 (1.57, 11.84)	0.005
	No	1*			1*		
	Smoking status						
	Yes	3.17 (0.14)	2.52 (0.48,13.15)		5.11 (0.04)	3.02 (0.53, 17.2)	0.21
	No	1*			1*		
	Gender						
	Male	1.47 (0.19)	1.07 (0.56,2.07)	0.82	1.92 (0.13)	1.08 (0.422, 2.76)	0.87
	Female	1*			1*		
	Age						
	≥ 50	1.07 (0.21)	0.89 (0.47,1.67)	0.72	2.39 (0.04)	1.95 (0.5,4.18)	0.02
	< 50	1*			1*		
	BMI						
	≥ 25 kg/m^2^	7.18 (0.001)	5.9 (1.94,18.06)	0.002	3.74 (0.17)	2.35 (0.704, 7.8)	0.17
	< 25 kg/m^2^	1*			1*		

*Note: p* < 0.05 statistically significant,1*= Reference category, AOR= adjusted odds ratio, COR= crude odds ratio.

Physical activity, alcohol consumption, age, and BMI were independent variables significantly linked to fasting blood glucose levels. Hypertensive individuals with low physical activity were 1.4 times more likely to have hyperglycemia. In addition, hypertensive patients who consumed alcohol were more likely to have both hyperglycemia and hypertriglyceridemia. Older and obese hypertensive patients were more likely to have both hyperglycemia and hypertriglyceridemia. (Table 8).

**Table 8:** Logistic regression analysis for factors associated with abnormal hyperglycemia and triglyceridemia results at Wallaga University Referral Hospital from June 2 to August 20, 2022.

Variables		Dependent variables					
		Glucose			Triglycerides		
		COR	AOR (95% CI)	*p* value	COR	AOR (95% CI)	*p* value
Independent variables	Physical activity						
	Low	1.87 (0.01)	1.4 (1.02, 3.08)	0.04	0.46 (0.17)	1.94 (1.25, 3.34)	0.99
	High	1*			1*		
	Alcohol drinking						
	Yes	4.24 (0.001)	4.33 (1.56, 12.03)	0.005	4.52 (0.001)	4.08 (1.55, 10.72)	0.004
	No	1*			1*		
	Smoking status						
	Yes	1.68 (0.05)	1.14 (0.164, 12)	0.89	1.17 (0.13)	2.735 (0.127,4.2)	0.07
	No	1*			1*		
	Gender						
	Male	1.94 (0.07)	1.49 (0.67,3.3)	0.32	1.42 (0.22)	1.05 (0.57,1.94)	0.06
	Female	1*			1*		
	Age						
	≥ 50	1.44 (0.03)	2.3 (0.132,4.68)	0.004	1.87 (0.18)	2.71 (0.38,4.23)	0.26
	< 50	1*			1*		
	BMI						
	≥ 25 kg/m^2^	3.85 (0.008)	2.95 (1.02, 9.06)	0.05	6.38 (0.002)	5.09 (1.54,16.88)	0.008
	< 25 kg/m^2^	1*			1*		

*Note: p* < 0.05 statistically significant, 1*=Reference category, AOR= adjusted odds ratio, COR= crude odds ratio.

Hypertensive individuals who were obese were 12.07 times more likely to have elevated LDL‐C levels compared to those with a normal weight (AOR = 12.07; 95% CI = 3.9–19.2) (Table 9).

**Table 9:** Logistic regression analysis for factors associated with abnormal HDL‐C and LDL‐C results among hypertensive patients at Wallaga University Referral Hospital from June 2 to August 20, 2022.

Variables		Dependent variables					
		HDL			LDL		
		COR (P‐value)	AOR (95% CI)	*p* value	COR (*p* value)	AOR 95% CI	*p* value
Independent variables	Physical activity						
	Low	1.65 (0.06)	1.69 (0.67, 4.24)	0.26	1.5 (0.10)	1.35 (0.34,5.45)	0.66
	High	1*			1*		
	Alcohol drinking						
	Yes	3.03 (0.022)	2.67 (0.05, 7.8)	0.06	2.06 (0.12)	1.23 (0.38,3.97)	0.72
	No	1*			1*		
	Smoking status						
	Yes	1.77 (0.05)	1.315 (0.06,1.65)	0.17	0.72 (0.02)	2.512 (0.044,5.9)	0.59
	No	1*			1*		
	Gender						
	Male	1.37 (0.03)	1.203 (0.67, 2.16)	0.56	0.57 (0.13)	1.5 (0.66,3.41)	0.33
	Female	1*			1*		
	Age						
	≥ 50	1.46 (0.17)	1.402 (0.79,2.74)	0.25	2.65 (0.23)	1.52 (0.23,3.16)	0.11
	< 50	1*			1*		
	BMI						
	≥ 25 kg/m^2^	0.47 (0.17)	1.56 (0.508, 4.79)	0.44	12.2 (0.001)	12.07 (3.9,19.2)	< 0.001
	< 25 kg/m^2^	1*			1*		

*Not*e: *p* < 0.05 statistically significant, 1*=Reference category, AOR= adjusted odds ratio, COR= crude odds ratio.

## 5 Discussion

This study assessed fasting blood glucose, electrolytes, creatinine, uric acid, and lipid levels in both hypertensive and non‐hypertensive participants. The results showed that fasting blood sugar and triglycerides were notably elevated in the hypertensive group compared to the non‐hypertensive group. Conversely, levels of sodium, creatinine, uric acid, total cholesterol, LDL‐C, and HDL‐C were significantly lower in the hypertensive participants compared to their non‐hypertensive counterparts.

In this study, the fasting blood glucose level of hypertensive participants was significantly higher, with a mean of 122.6 ± 49.5, compared to the non‐hypertensive group, which had a mean of 102.6 ± 29.56, with a *p*‐value of 0.001 at a 95% confidence interval. These findings are consistent with previous studies conducted in Korea, China, and India [24, 25, 26]. This was attributed to hypertension causing microvascular dysfunction, which may play a role in the development of diabetes. Additionally, endothelial dysfunction, which is linked to insulin resistance, is closely connected to hypertension, and biomarkers of endothelial dysfunction have been identified as independent predictors of hyperglycemia [27, 28].

In this study, serum creatinine and uric acid levels were higher in the non‐hypertensive participants, with means of 0.88 ± 0.49 and 4.8 ± 1.6, respectively, compared to the hypertensive group, which had means of 0.83 ± 0.33 and 4.73 ± 1.2. These results differ from findings in previous studies conducted in India and Cameroon [29, 30]. This could be attributed to advancing glomerular damage, endothelial dysfunction, and renal microvascular disease [26]. Lower creatinine among hypertensive patients may be due to antihypertensive treatment, which slows the progression of kidney damage and preserves glomerular filtration rate [31]. In addition, a reduced purine‐rich diet and alcohol consumption, as a result of diet modification consultation for hypertensive patients, may reduce uric acid [32].

Serum sodium levels were notably lower in hypertensive participants, averaging 134.5 ± 11.9, compared to non‐hypertensive participants, who had an average of 138.7 ± 5.1, with a *p*‐value of < 0.001 at a 95% confidence interval. The possible reason for low serum sodium in hypertensive patients may be due to diuretic drug therapy that induces urinary excretion of sodium. In addition, reduced dietary sodium intake as a part of lifestyle modification may be another possible reason for low serum sodium [33, 34], while there was no significant difference in serum potassium levels between the study groups, which is consistent with findings from previous studies conducted in Nigeria [35].

Total cholesterol, LDL‐cholesterol, and HDL‐C levels were significantly higher in non‐hypertensive participants, with averages of 185.2 ± 49.7, 120 ± 44.9, and 51.4 ± 51.3, respectively, compared to hypertensive participants, who had averages of 169.2 ± 45.3, 104.6 ± 42.8, and 30.3 ± 24.7. The *p*‐values were 0.02, 0.01, and 0.001 at a 95% confidence interval, respectively. The possible justification for decreased total cholesterol, decreased LDL‐c, and increased HDL‐c may be due to lipid‐lowering therapy and lifestyle modification practices [36]. Additionally, triglyceride levels were lower in non‐hypertensive participants, with a mean of 151.7 ± 49.4, compared to hypertensive participants, who had a mean of 179.3 ± 58, with a *p*‐value of 0.001 at a 95% confidence interval. These findings are consistent with previous studies conducted in India and Bangladesh [37, 38].

For hypertensive patients with a hypertension duration exceeding 5 years, fasting blood glucose, creatinine, uric acid, total cholesterol, and LDL‐cholesterol levels were significantly higher, with means of 163.91 ± 46.01, 0.96 ± 0.37, 5.02 ± 0.83, 209.98 ± 61.13, and 129.02 ± 37.72, respectively. These values were notably higher compared to those in patients with hypertension lasting less than 5 years, who had means of 123.96 ± 52.1, 0.78 ± 0.31, 4.41 ± 0.98, 169.20 ± 46.24, and 108.75 ± 43.53, with p‐values of 0.001, 0.022, 0.007, 0.001, and 0.042 at a 95% confidence interval. Additionally, sodium levels were significantly lower in patients with hypertension for less than 5 years. No significant differences were observed in triglycerides and HDL‐C based on the duration of hypertension.

Factors associated with abnormal Clinical Chemistry findings in this study were: overweight (obese), age, physical activity, smoking, and alcohol drinking in hypertensive patients. The present study shows that being overweight (obesity) was significantly associated with serum glucose, triglycerides, total cholesterol, and LDL‐C. A person who was overweight (obese) had hyperglycemia, hypercholesterolemia, and elevated LDL‐C.

Body mass index was an independent variable significantly associated with serum Total Cholesterol. Hypertensives were obese (5.9) times more likely to have hypercholesterolemia than normal weight (AOR = 5.9; 95% CI = 1.94–18.06). Obese hypertensive individuals were obese (12.07) times more likely to have elevated LDL‐C levels than normal weight (AOR = 12.07; 95% CI = (3.9–19.2). Hypertensive individuals who consumed alcohol were (4.08), and obese individuals were (5.09) times more likely to have hypertriglyceridemia than non‐alcoholic and normal weight (AOR = 4.08; 95% CI = 1.55–10.72, AOR = 5.09; 95% CI = 1.54–16.88), respectively. The study conducted in Algeria in hypertensive patients, similar to this finding, detailed that subjects with overweight/obesity had significantly higher TC, LDL‐C, TG, and lower HDL‐C [39]. This may be attributed to metabolic abnormalities associated with being overweight or obese. In this study, age, physical activity, and alcohol consumption were independent factors significantly related to serum glucose levels in hypertensive patients. Older hypertensive individuals were 2.3 times more likely to have elevated glucose levels compared to younger patients (AOR = 2.3; 95% CI = 0.132–4.68). Alcohol consumers were 4.33 times more likely to have elevated glucose levels compared to non‐drinkers (AOR = 4.33; 95% CI = 1.56–12.03), while those with low physical activity levels were 1.4 times more likely to have elevated glucose compared to those with high physical activity (AOR = 1.4; 95% CI = 1.02–3.08). Age was also a significant independent factor associated with serum sodium levels; hypertensive patients aged> 50 were 1.95 times more likely to experience hypernatremia than younger patients (AOR = 1.95; 95% CI = 0.5–4.18). Age‐related declines in glomerular filtration rate are common in the elderly, accompanied by reduced renal blood flow, decreased kidney mass, particularly in the renal cortex, changes in glomerular hemodynamics, and increased permeability of the glomerular basement membrane [2, 40].

Lifestyle changes such as weight loss, regular exercise, and a healthy diet are effective predictors of reduced blood pressure and lower risk of hypertension. For instance, the Canadian Hypertension Education Program guidelines (2016) recommend health behavior management for hypertension prevention, including physical exercise, weight control, reduced alcohol consumption, stress management, and decreased sodium intake. They also suggest dietary modifications to increase potassium intake through whole foods rather than supplements (for patients not at risk of hyperkalemia), as this approach provides additional nutritional benefits [41].

### 5.1 Limitations of the Study

The study conducted was a cross‐sectional study, which cannot provide adequate information about the causal relationship between dependent variables and risk factors. It did not thoroughly assess dietary habits. Additionally, it was challenging to accurately determine the exact amount of salt added to the diet and how much salt was present in various foods. Moreover, hypertensive and non‐hypertensive groups differed significantly in age, and residual confounding by age may have influenced some of the observed biochemical differences. Regardless of these limitations, this study offers valuable insight into the scarce data situation of Ethiopia.

## 6 Conclusion

Hypertensive patients had significantly higher levels of fasting blood sugar and triglycerides compared to those without hypertension. Conversely, serum sodium, creatinine, uric acid, total cholesterol, LDL‐C, and HDL‐C levels were significantly lower in the hypertensive group compared to the non‐hypertensive group.

### 6.1 Recommendation

Hypertensive patients require regular follow‐up that includes fasting blood glucose, serum electrolytes, lipid profiles, and renal function tests to monitor and prevent chronic complications. Consistent monitoring of these laboratory parameters is essential for managing hypertension. Additionally, further research with a broader range of parameters and across different study locations is needed to enhance our understanding of these parameters in hypertension.

## Author Contributions

**Amanuel Hika:** conceptualization, investigation, funding acquisition, writing – original draft, methodology, writing – review and editing, software, formal analysis. **Sintayehu Asaye Biya:** conceptualization, funding acquisition, validation, methodology, writing – review and editing, data curation, project administration, supervision, software, visualization. **Temam Ibrahim Husen:** conceptualization, investigation, funding acquisition, methodology, validation, visualization, writing – review and editing, formal analysis, software, supervision. **Negesse Bokona Rufe:** investigation, funding acquisition, methodology, validation, visualization, writing – review and editing, formal analysis, software, data curation, supervision. **Hundesa Emana Urgesa:** conceptualization, investigation, validation, visualization, formal analysis, data curation, supervision, software. **Bedasa Addisu:** conceptualization, investigation, funding acquisition, writing – original draft, visualization, writing – review and editing, methodology, software, formal analysis, data curation, supervision, resources.

## Ethics Statement

Ethical clearance with reference number IHRPG0/851 was obtained from the institutional review board of the Health Sciences faculty, Jimma University. A letter of cooperation was written from the Jimma University research coordination office to Wallaga University Referral Hospital. All methods to conduct this study were performed in accordance with the relevant guidelines and regulations in the Helsinki Declaration. Written informed consent was obtained from all study participants, and permission was obtained from a legal guardian for illiterate participants. The purpose, benefit, and method of the study were clearly explained to the participants, and the participants were informed that their responses would be kept confidential. Participation in the study is voluntary, and refusal is possible.

## Conflicts of Interest

The authors declare no conflicts of interest.

## Transparency Statement

Bedasa Addisu affirms that this manuscript is an honest, accurate, and transparent account of the study being reported; that no important aspects of the study have been omitted; and that any discrepancies from the study as planned have been explained.

## Data Availability

The data that support the findings of this study are available from the corresponding author upon reasonable request.
